# The Rough Guide to In Silico Function Prediction, or How To Use Sequence and Structure Information To Predict Protein Function

**DOI:** 10.1371/journal.pcbi.1000160

**Published:** 2008-10-31

**Authors:** Marco Punta, Yanay Ofran

**Affiliations:** 1 Department of Biochemistry and Molecular Biophysics, Columbia University, New York, New York, United States of America; 2 Columbia University Center for Computational Biology and Bioinformatics (C2B2), New York, New York, United States of America; 3 Northeast Structural Genomics Consortium (NESG), Columbia University, New York, New York, United States of America; 4 The Mina and Everard Goodman Faculty of Life Sciences, Bar-Ilan University, Ramat-Gan, Israel; Whitehead Institute, United States of America


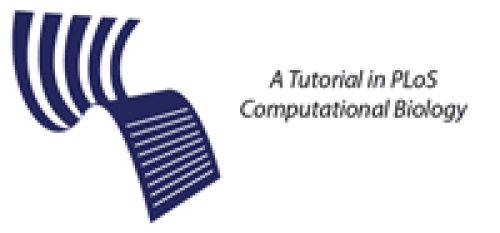


## Introduction

### 

#### Choosing the right function prediction tools

The vast majority of known proteins have not yet been characterized experimentally, and there is very little that is known about their function. New unannotated sequences are added to the databases at a pace that far exceeds the one in which they are annotated in the lab. Computational biology offers tools that can provide insight into the function of proteins based on their sequence, their structure, their evolutionary history, and their association with other proteins. In this contribution, we attempt to provide a framework that will enable biologists and computational biologists to decide which *type* of computational tool is appropriate for the analysis of their protein of interest, and what kind of insights into its function these tools can provide. In particular, we describe computational methods for predicting protein function directly from sequence or structure, focusing mainly on methods for predicting molecular function. We do not discuss methods that rely on sources of information that are beyond the protein itself, such as genomic context [1], protein–protein interaction networks [2], or membership in biochemical pathways [3]. When choosing a tool for function prediction, one would typically want to identify the best performing tool. However, a quantitative comparison of different tools is a tricky task. While most developers report their own assessment of their tool, in most cases there are no standard datasets and generally agreed-upon measures and criteria for benchmarking function prediction methods. In the absence of independent benchmarks, comparing the figures reported by the developers is almost always comparing oranges and apples (for discussion of this problem see [4]). Therefore, we refrain from reporting numerical assessments of specific methods. For those cases in which independent assessment of performance is available, we refer the reader to the original publications. Finally, we discuss only methods that are either accessible as Web servers or freely available for download (relevant Web links can be found in Table S1).

#### What is protein function?

The first problem we face when dealing with protein function is well-illustrated by the title of a 1998 article by Schubert et al. [5], “The X-ray structure of a cobalamin biosynthetic enzyme, cobalt-precorrin-4 methyltransferase.” What is the function of the protein that is described in this paper? The authors report the solution of the crystal structure of CbiF, which is an enzyme implicated in the biosynthesis of vitamin B12 (cobalamin). More specifically, CbiF transfers a methyl group from an S-adenosyl-L-methionine molecule to a precursor of vitamin B12 (cobalt-precorrin-4). Vitamin B12 is a compound that “helps maintain healthy nerve cells and red blood cells, and is also needed to make DNA” [6]. Its deficiency is related to anemia, as well as to several neurological and psychiatric symptoms [7]. As we see, CbiF function comes in different flavors: molecular/enzymatic (methyltransferase), metabolic (cobalamin biosynthesis—directly—and DNA biosynthesis—indirectly), and physiological (maintenance of healthy nerve and red blood cells, through B12), along with possible consequences related to their malfunctioning. There are, obviously, numerous ways to describe each of these aspects of the protein function. Enzymatic function, for example, may be characterized through: reaction (methylation), substrate (cobalt-precorrin-4), or ligand (S-adenosyl-L-methionine).

#### Classifying and predicting

Since protein function has many facets, its prediction has different meaning for different people. It may mean the prediction of the cellular process in which the protein is involved, or the nitty-gritty of its enzymatic activity, or rather its physiological role. Therefore, when attempting to predict protein function one should first define clearly the kind of function she or he wants to predict. When predicting function automatically on a large scale, this problem is intensified by the need to standardize and quantitatively assess the similarity of functions between proteins. While defining sequence and structural similarity may be easy, there is no a priori straightforward measure we can use to put a number on the similarity of functions between two proteins. Prediction methods could not be developed, or rigorously assessed, without such measure. Several large-scale projects attempted to respond to this challenge by building classification systems or ontologies of biological functions (see [8],[9] for review). One such enterprise was launched as early as 1955 by the International Congress of Biochemistry, which created the Enzyme Commission to come up with a nomenclature for enzymes. In this numerical classification, each enzymatic function could be described by a set of four numbers (which, together, are dubbed EC number). Each of these four numbers represents specific description of the enzyme and its activity. For instance, when comparing carboxylesterase (3.1.1.1) and isochorismatase (3.3.2.1), one can tell that they share the basic enzymatic activity of a hydrolase (all hydrolases have 3 as the first number), but they act on different types of bonds: hydrolases with 3.1.-.- act on an ester bond and those with 3.3.-.- act on an ether bond. This system is infinitely expandable to include any new enzyme, but it does not cover functions that are not enzymatic. The Gene Ontology (GO) project provides a controlled vocabulary to describe the function of any gene product in any organism. It developed three structured controlled vocabularies to cope with the multifaceted nature of the biological function. For each gene product, GO can provide a number for its cellular component, the biological process in which it is involved, and its specific molecular function. Various algorithms have been proposed to assign a score for the similarity between numbers within each of these three ontologies [10],[11]. Thus, GO has become the standard for assessing the performance of function prediction methods.

## Function Annotation Transfer from Sequence

### 

#### Homology useful but different from “same function”

**Figure 1:**
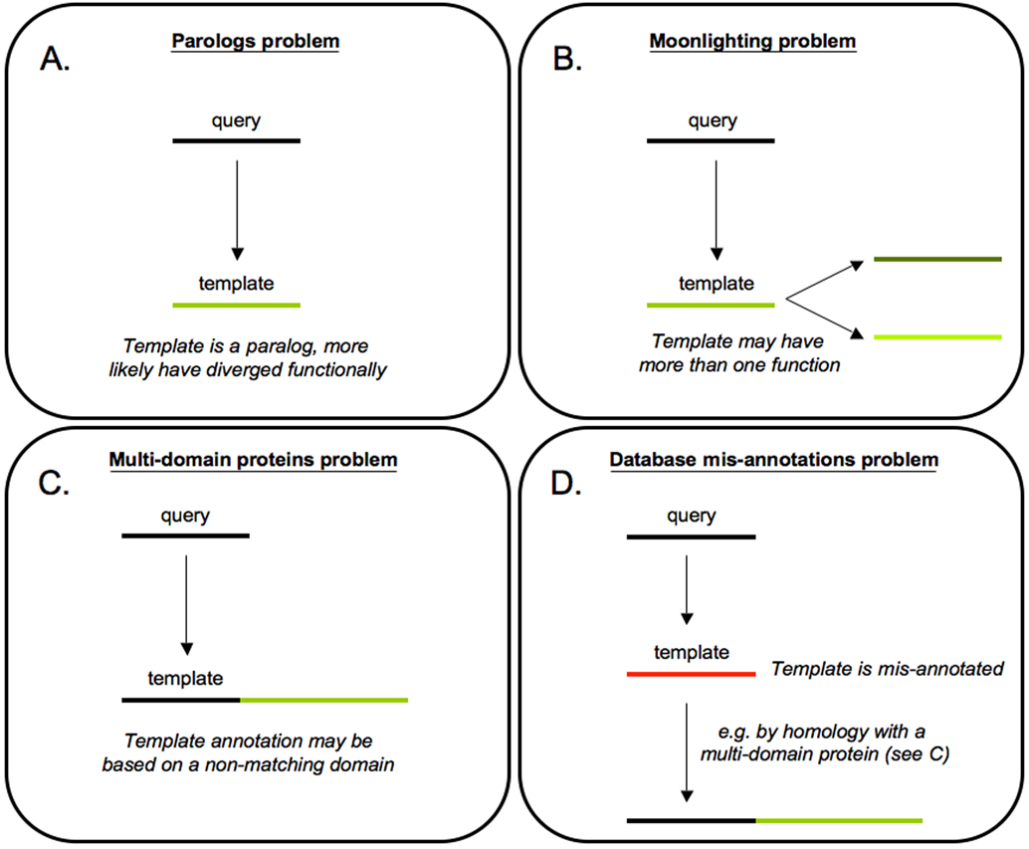
Homology based annotation transfer: Problems. (A) Paralogy problem: Paralogs are more likely to diverge functionally with respect to orthologs. If our putative template is a paralog, the probability that the query has similar function decreases. (B) Moonlighting problem: If the template performs multiple functions, the query could have retained only some of them (and vice-versa, if the query were a moonlighting protein, using a non-moonlighting template would result in an incomplete annotation of the query). (C) Multi-domain proteins problem: If the template is annotated based on the function of a domain that is not aligned to the query, annotation transfer is not possible. (D) Database mis-annotations problem: Database entries may have been mis-annotated; the risk is especially high if annotation was performed automatically via homology transfer.

The most widely used approach for function prediction is homology transfer. Given an unannotated protein, this approach suggests searching for an annotated homolog and using the experimentally verified function of the latter to infer the function of the former. However, this procedure should be implemented with caution. Homology is often confused with similarity of function. In reality, homology between two proteins simply means that they have a common evolutionary origin. Whether or not they have since retained similarity in any of their properties is something that needs to be checked in each individual case. An important distinction in this context is between orthologous and parologous sequences: orthologs are genes that originated from a common ancestor through a speciation event, while paralogs are the results of duplication events within the same genome. In general, function tends to be more conserved in orthologs than in paralogs [12]. So, when attempting to predict the function of an unannotated protein based on its homology to an annotated one, one should search for orthologs rather than paralogs (Figure 1A). Although several databases have been created to help identify orthologous genes (e.g., COGs [13] and InParanoid [14]), “proven orthologs are as rare in the literature as diamonds in bare rock” [12]. Orthologs, additionally, may also diverge functionally, sometimes more than corresponding paralogs [12]. Finally, there exist functional similarities between proteins that are not reflected in homology. These facts underline the difficulty of the task of transferring function from a homologous template.

In practice, the most common way to infer homology is by detecting sequence similarity (note, however, that remote relationships will generally be missed by sequence similarity approaches; see the section about structure below). Popular sequence alignment methods include PSI-BLAST [15], HMMER [16], and SAM [17]. When investigating the function of a protein, we ought to align its sequence against a database of annotated proteins, such as SWISS-PROT [18], in order to find its homologs of known function. The question we need to address is how two homologous proteins relate functionally. As we mentioned previously, several studies have shown that homology (both orthology and paralogy) does not guarantee conservation of function (Table 1). Indeed, relatively small differences in sequence can sometimes cause quite radical changes in functional properties, such as a change of enzymatic action, or even a loss or acquisition of the enzymatic activity itself. It is also apparent that there is no sequence similarity threshold that guarantees that two proteins share the same function (see references in Table 1). Thus, although higher sequence similarity increases confidence in function annotation transfer, there is no threshold that can be considered safe. An extreme case is represented by the so-called “moonlighting proteins” or proteins that perform multiple and, at times, significantly different functions [19],[20]. For example, η-crystallin is a protein that plays a structural role in the eye lens of several species, while working as an enzyme in other tissues. Homologs of these proteins may retain only some of the original functions [21]. As a consequence, function annotation transfer may result in erroneous or incomplete assignments (Figure 1B).

**Table 1:** Do's and Don'ts of annotation transfer by homology.

Functional property to be conserved	Sequence identity	Conservation rate	Reference
Non-enzyme	50%	98%*	[88]
All 4 EC numbers	70%**	90%	[89]
All 4 EC numbers	40%**	70%	[89]
First 3 EC numbers	50%**	90%	[89]
First 3 EC numbers	30%**	70%	[89]
All 4 EC numbers	50%	30%	[90]
First 3 EC numbers	25%	70%	[91]
SWISS-PROT keywords	40%	70%	[92]
Subcellular localization (11 classes)	70%	90%	[93]

*: 98% of non enzymes that have at least one enzyme homolog.

**: Global identity, defined in [89].

Note: different estimates for the same functional aspects reflect the different methods, procedures, and datasets used to assess sequence similarity by the various groups.

The multi-domain nature of many proteins can also be the cause of annotation transfer errors (Figure 1C). In fact, in databases storing entire sequences (such as SWISS-PROT [18]), functional annotation of a protein may refer to any of its domains. If the query protein (i.e., the protein whose function we wish to predict) does not align to that specific domain, annotation transfer is totally unjustified and will very likely result in a mis-annotation. While a number of databases and tools attempt to split proteins into domains based on sequence (Pfam [16], PRODOM [22], SMART [23]), the most reliable way to identify protein domains is by using, when possible, structural knowledge (SCOP [24], CATH [25]).

Some of these problems can be mitigated by the use of phylogenomic inference that frames sequence evolutionary relationship into a phylogenetic context as described in [26].

To complicate matters further, bear in mind that databases contain incorrect annotations, mostly caused by erroneous automatic annotation transfer by homology [27] (Figure 1D). Thus, always check the source of the annotation before you use it.

In conclusion, homology between two proteins does not guarantee that they have the same function, not even when sequence similarity is very high (including 100% sequence identity) (Table 2). Bottom line: when annotating function, you won't get too far with the classic 25%–30% sequence identity that is so powerful for structure prediction. On the positive side, the higher the sequence similarity the better the chance that homologous proteins in fact share functional features (Tables 1 and 2). As we have seen, correct transfer of functional annotation from a protein to its homolog depends on whether the two proteins are orthologs or paralogs, on the level of sequence similarity, on the type of annotation we want to transfer (for example, prediction of subcellular localization typically requires lower sequence identity than prediction for enzymatic function [28]), and on the specific domain aligned. No sequence similarity threshold is safe for blind annotation transfer.

**Table 2:** Do's and Don'ts of annotation transfer by homology.

			Yes	No
Homology	=	Same function		√
Orthology	=	Same function		√
Paralogy	=	Same function		√
Orthology	=	>Probability of same function	√	
Paralogy	=	<Probability of same function	√	
Same sequence	=	Same function		√
Sequence similarity>threshold	=	Same function		√
Homology+conservation of functional residues	=	Same function		√
Similar structure	=	Similar function		√
>Sequence similarity	=	>Probability of same function	√	
>Structure similarity	=	>Probability of same function	√	

#### Sequence signatures predict functional traits

In some cases, a relatively small sequence signature may suffice to conserve the function of a protein even if the rest of the protein has changed considerably during the course of evolution. Alternatively, non-homologous proteins could acquire the same functional motif independently (convergent evolution). Thus, two proteins that would not find each other in a sequence search may still have common sequence signatures that could surrender their functional relatedness. Clearly, if two proteins have some level of overall sequence similarity and also share a common motif, the confidence of annotation transfer increases. Several computational tools are dedicated to the identification of functional motifs (e.g., PRINT-S [29], BLOCKS [30], PROSITE [31], InterPro [32], and ELM [33]). They usually offer a large library of sequence motifs that have been collected either manually by experts, or automatically by pattern-searching algorithms, or by a combination of the two. When a query sequence is submitted to these tools, it is compared to all known motifs in search of a match. Finding one of these well-characterized motifs in a newly discovered sequence could offer some insights into its function.

More generally, residues that are crucial for the function of the protein can often be identified through the use of multiple sequence alignments that highlight conservation patterns in protein families (see [34] and [35] for more detailed discussion of these methods). This approach is possible, of course, when multiple homologs of the protein of interest are available. Importantly, even when the function of specific conserved residues within the protein family is not known, multiple sequence alignments point to regions that may be of interest for experimental functional characterization (e.g., by means of site directed mutagenesis). Multiple sequence alignments are also relevant as input to methods that map sequence conservation on the protein surface (see below).

## Function Annotation Transfer from Structure

### 

#### Structure better than sequence alone

**Figure 2:**
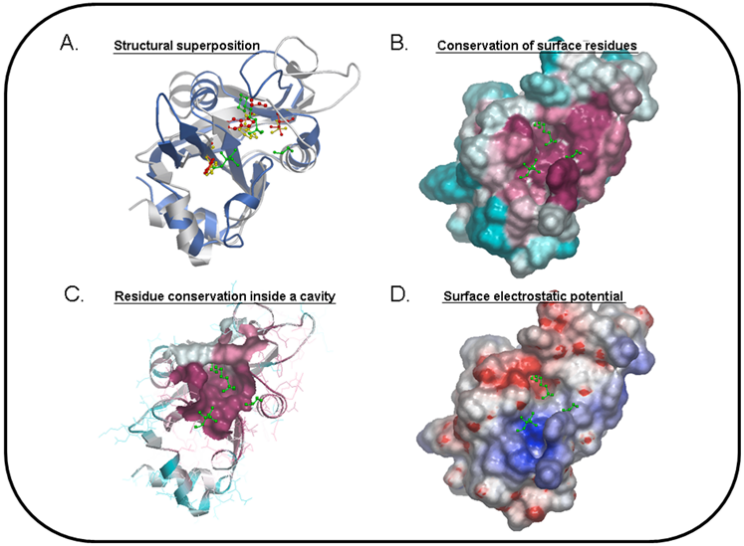
Using structure to predict function. The protein represented here is PDBid: 2eve. All figures are derived from the Northeast Structural Genomics Consortium structure gallery (http://nmr.cabm.rutgers.edu:9090/gallery/jsp/Gallery.jsp). AstexViewer 2.0 [49] is used for visualization. (A) Superposition of 2eve structure (gray) and of the structure of a homolog (blue, PDBid: 2ar1), using Skan [59]. 2eve hosts three co-crystallized small non-functional ligands (green; ball and stick). Three structurally aligned residues of 2eve and 2ar1 are also shown (red and yellow; ball and stick). (B) Surface residue conservation: Conserved residues (mauve) versus variable residues (cyan). Conservation is calculated as follows: homologs of 2eve are collected using three iterations of PSI-BLAST [15] retaining all homologs with E-value<10−3 and reducing redundancy at 80% sequence identity with CD-HIT [85]. Then, a multiple sequence alignment is created using CLUSTALW [86]. Finally, the multiple sequence alignment is used as input to ConSurf [54], which uses it to calculate residue conservation. (C) Residue conservation within the protein largest cavity (as defined by SCREEN [87]). (D) 2eve surface electrostatic potential (using GRASP2 [59]) (positive in blue, negative in red).

Proteins live and function in 3D, and therefore structural information is very helpful for predicting function. The need for tools to predict function from structure is intensified by the success of the structural genomics enterprises that deposit hundreds of new experimentally solved structures of proteins with unknown function [36]. Structural information, however, does not have to come directly from the protein of interest but can also be derived from a homologous protein via modeling [37]. Unfortunately, as with sequence, two proteins having the same overall structural architecture, and even conserved functional residues [38], can have unrelated functions. Additionally, two proteins can perform the same function while having radically different structures [39]. Still, structure may help function prediction in several ways. Structural similarity between two proteins may reveal their common evolutionary origin even in the absence of significant sequence similarity, possibly suggesting similar function (Figure 2A). Or, it may indicate evolutionary convergence caused by common functional constraints. Prokaryotic virulence effectors offer some remarkable examples of functional convergence. Some of these proteins, in order to be able to tamper with the biological processes of the host, have adapted to mimic host proteins. This is achieved by either mimicking their overall architecture or, more often, their local structural features [40],[41]. Numerous methods have been developed to perform structural comparisons, using the Protein Data Bank [42] or structure classification databases (SCOP [24], CATH [25]) as a source. Among the most used structural alignment methods are SSM [43], FATCAT [44], DALI [45], and CATHEDRAL [46] (see [47] for a comparison of the performance of several methods). In general, it is suggested to use more than one method since different methods may capture different valid matches. Most programs provide a PDB-type output file for the two aligned proteins that can be uploaded to one of the many available structure visualization programs (e.g., VMD [48], AstexViewer 2.0 [49]). When evaluating the functional implications of a match, we need to consider how functionally promiscuous a given structural architecture is (i.e., whether or not it is known to relate to many functions [50]), and we have to check the conservation of functional residues. Functional residues may not be perfectly conserved in proteins of similar function. In fact, specific residues may be responsible for different ligand or substrate binding affinities or for different reaction rates in enzymes. However, disruption of the 3D core of an active site in an overall conserved structural architecture should be a serious concern [51]. Catalytic Site Atlas [52] and MACiE [53] are databases where you can find detailed information about functional residues and their specific role in enzymes.

Even in the absence of a structurally related protein, structure may provide important functional information by highlighting properties of the protein's accessible surface that may relate to function. These include residue conservation (Consurf [54], siteFiNDER|3D [55], TRACE [56], Figure 2B), cavities (CASTp [57], Q-SiteFinder [58], Figure 2C), and electrostatic patches (GRASP2 [59], Figure 2D). In general, structural knowledge, although not a panacea for all problems, is an extremely powerful tool for computational function prediction.

#### Structural motifs reveal binding sites

The idea is similar to sequence motifs: functional aspects may be defined by local structural signatures. Residues found in functional signatures may be not be adjacent in sequence; however, they do tend to cluster in the 3D structure, forming binding sites for ions, small molecules, DNA, RNA, or other proteins. There are databases and tools for searching such structurally defined motifs in a structure of interest (JESS [60], RIGOR [61], PAR-3D [62], PINTS [63], and PDBSiteScan [64]). As usual, the effectiveness of such methods depends on the specific function being predicted and on the desired level of detail of the prediction.

## De Novo Function Prediction Using Sequence and Structure

### 

#### De novo predictions push the limit

What can we do when the protein whose function we want to predict has no significant similarity to any annotated protein? Several approaches have been suggested to predict protein function de novo. That is, using sequence or structure information without relying on similarity to a specific protein but rather on the “generic” properties that are common to proteins of the same function. Indeed, proteins of the same function have to adapt to similar constraints (e.g., pH, properties of a ligand, structural flexibility), which will be reflected in their sequence and structural features. De novo methods are generally based on machine learning algorithms that are able to capture significant non-trivial correlations between features and functions. These methods are usually less accurate than annotation transfer but enjoy higher coverage, eventually protruding into experimentally yet unexplored regions of the sequence space and allowing annotation of entire genomes. Hereafter, we report on some of the most successful de novo methods.

#### Functional residues

Residues that have similar function in different proteins are likely to possess similar physicochemical characteristics. For example, residues that bind DNA share common structural and physicochemical features in most DNA-binding proteins (e.g., secondary structures, geometries, solvent accessibility, charge, hydrophobicity). Once these features are characterized and quantified, it may be possible to search for residues that possess them, thus predicting their function. There are several methods for the prediction of DNA binding residues from sequence (e.g., DISIS [65] and bindN [66]) or structure (e.g., Patchfinder+ [67]). Another example is represented by residues that bind metals. The number and type of residues binding to a given metal may considerably differ from protein to protein. For this reason, known sequence metal binding motifs are useful but cover only a small fraction of all binding sites [68]. Recently, de novo methods have been developed that specialize in predicting metal binding sites from sequence (MetalDetector [69]) and from structure (MetSite [70] and CHED [71]), the latter exploiting successfully the tight clustering of metal binding residues in 3D.

#### Subcellular localization

Knowing the subcellular localization of a protein helps to narrow down the number of functions the protein can perform and can be very relevant for its experimental characterization [72]. Subcellular localization can be predicted from homology and motifs, with the aforementioned limitations. De novo methods, instead, exploit the known correlation between amino acid composition and localization [73]. LOCtree [74], BaCelLo [75], TARGETp [76], Protein Prowler [77], and the PSORT suite of programs [78]—some combining de novo, homology, and motifs—are among the best methods available.

#### Programs that predict function combining different sources of information

Another, more ambitious, approach is to integrate various aspects of proteins and to try to associate them with specific GO numbers. Since protein function is a multifaceted notion, its comprehensive prediction requires data from many sources. Thus, these methods attempt to integrate all sorts of information that pertain to function such as structure, sequence information, physicochemical features, and even protein interaction data. Such an approach is taken, for example, by ProtFun [79], which combines 14 different sequence-based prediction methods such as prediction of glycolization sites, number of negative and positive residues, predicted transmembrane helices, predicted subcellular localization, and other features, and integrates them to yield a GO term. ProKnow [80] relies predominantly on structural features that are associated with specific functions as well as on sequence motifs and interaction data. Similarly, ProFunc [81] uses structure and sequence motifs, combined with identification of active and binding sites and integrates them with interaction data and knowledge of genomic sequences to yield a comprehensive prediction of function.

Several more de novo methods that are relevant for function exist, including predictors of coil-coiled regions [82], natively unstructured regions [83], and post-translational modifications [84].

## Supporting Information

Table S1 Publicly available tools.
(0.18 MB DOC)
