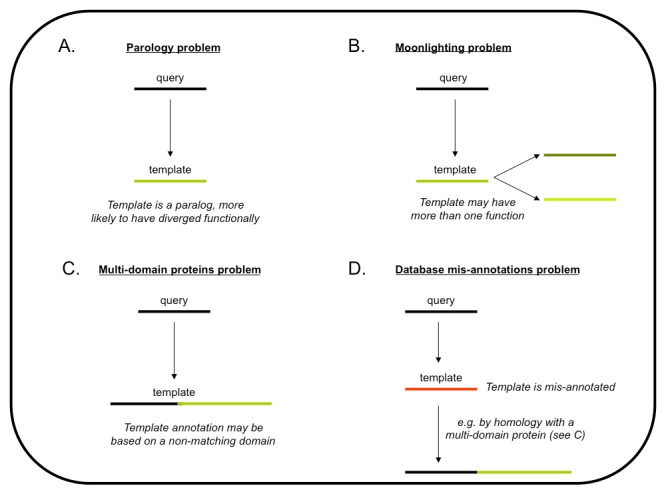# Correction: The Rough Guide to In Silico Function Prediction, or How To Use Sequence and Structure Information To Predict Protein Function

**DOI:** 10.1371/annotation/3d8d748f-d1be-4ba9-84b3-23d2a582338b

**Published:** 2008-11-10

**Authors:** Marco Punta, Yanay Ofran

Figure 1A has a mistake in the text. The correct version of Figure 1 can be found here:

**Figure:**