# Supplementary material for: The Rough Guide to In Silico Function Prediction, or How To Use Sequence and Structure Information To Predict Protein Function
Source: PLoS Comput Biol. 2008 Oct 31;4(10):e1000160. doi: 10.1371/journal.pcbi.1000160 (PMC2518264; doi:10.1371/journal.pcbi.1000160)
Supplement: Table S1 — Publicly available tools. (0.18 MB DOC) [file pcbi.1000160.s001.doc]

Supplementary Material for: The rough guide to *in silico* protein function prediction

**Table ST1: Publicly available tools.**

| **Cornerstones** | | |
| --- | --- | --- |
| *URL* | *Service name* | *Additional info* |
| <http://www.rcsb.org/pdb/home/home.do> | PDB | Repository of all publicly available protein, DNA and RNA structures |
| <http://www.expasy.ch/sprot/> | Swiss-Prot | Most comprehensive database with a high level of functional annotation |
| **Ontologies and nomenclatures** | | |
| *URL* | *Service name* | *Additional info* |
| [http://www.geneontology.org](http://www.geneontology.org/) | GO | Gene Ontology |
| http://www.chem.qmul.ac.uk/iubmb/enzyme/ | IUBMB Enzyme Nomenclature | Enzyme Nomenclature |
| <http://expasy.org/enzyme/> | Enzyme | Enzyme Nomenclature |
| **Orthologous group databases** | | |
| *URL* | *Service name* | *Additional info* |
| [http://www.ncbi.nlm.nih.gov/COG/](http://www.pubmedcentral.nih.gov/redirect3.cgi?&&auth=0MMl20my6BogF-pRuVAsZAf1CCHlvLYuKqbe17kP8&reftype=extlink&artid=222959&iid=4162&jid=13&FROM=Article|Body&TO=External|Link|URI&article-id=222959&journal-id=13&rendering-type=normal&&http://www.ncbi.nlm.nih.gov/COG/) | COGs | Clusters of Orthologous Groups |
| <http://inparanoid.sbc.su.se/cgi-bin/index.cgi> | InParanoid | Eukaryotic Ortholog Groups. |
| **Databases containing information about enzyme active sites** | | |
| *URL* | *Service name* | *Additional info* |
| <http://www.ebi.ac.uk/thornton-srv/databases/CSA/> | Catalytic Site Atlas |  |
| <http://www.ebi.ac.uk/thornton-srv/databases/MACiE/> | MACiE | Only enzymes of known structure |
| **Sequence search and alignment methods** | | |
| *URL* | *Service name* | *Additional info* |
| <http://www.ncbi.nlm.nih.gov/blast/Blast.cgi> | PSI-BLAST | Position specific iterative BLAST |
| [http://hmmer.janelia.org](http://hmmer.janelia.org/) | HMMER |  |
| <http://www.soe.ucsc.edu/compbio/HMM-apps/HMM-applications.html> | SAM-T | Suite of programs including pair-wise and multiple sequence alignments |
| ***References:*** Alignments: where we need to improve[1]. | | |
| **Multiple sequence alignment methods** | | |
| *URL* | *Service name* | *Additional info* |
| <http://www.igs.cnrs-mrs.fr/Tcoffee/tcoffee_cgi/index.cgi> | T-Coffee | Collection of tools including multiple sequence and structure alignments |
| <http://www.ebi.ac.uk/Tools/clustalw2/index.html> | ClustalW2 |  |
| <http://probcons.stanford.edu/> | PROBCONS |  |
| <http://www.ebi.ac.uk/muscle/> | MUSCLE |  |
| ***References:*** [2]  ClustalW2 is the most used multiple sequence alignment method but is it the most accurate? Find it out reading this review. [3] | | |
| **Protein families and domains; sequence motifs and sequences patterns** | | |
| *URL* | *Service name* | *Additional info* |
| <http://pfam.sanger.ac.uk/> | Pfam | Domains. Extensive manual annotation |
| <http://www.tigr.org/TIGRFAMs/> | TIGRFAMs | Domains. |
| <http://prodom.prabi.fr/prodom/current/html/home.php> | ProDom | Domains. Also uses structural information from SCOP |
| <http://smart.embl.de/> | SMART | Domains. Extensive manual annotation. Most useful to detect regulatory domains |
| <http://www.bioinf.manchester.ac.uk/dbbrowser/PRINTS> | PRINTS | Identifies multiple motifs that characterize a protein family |
| <http://blocks.fhcrc.org/> | BLOCKS | Multiple alignments that correspond to most highly conserved regions |
| <http://www.expasy.ch/prosite> | PROSITE | Domains, families and functional sites |
| <http://www.ebi.ac.uk/interpro/> | InterPro | Collection of several other databases |
| <http://www.ebi.ac.uk/Tools/InterProScan/> | InterProScan | Tool to query InterPro |
| <http://elm.eu.org/links.html> | ELM | Eukaryotic motifs |
| ***References:*** A review of the methods used to define domains and motifs and of the linked databases[4]. | | |
| **Structure classification databases** | | |
| *URL* | *Service name* | *Additional info* |
| <http://scop.mrc-lmb.cam.ac.uk/scop/> | SCOP | Mostly manual |
| <http://www.cathdb.info/> | CATH | Mostly automated |
| ***References:*** A recent assessment of the agreement between automated and manual protein structure classification[5]; Beyond the ‘fold’ concept[6]. | | |
| **Structure search and alignment methods** | | |
| *URL* | *Service name* | *Additional info* |
| <http://fatcat.burnham.org/> | FATCAT | Allows for domain rigid body-type of rearrangements |
| <http://ekhidna.biocenter.helsinki.fi/dali_server/> | DALI |  |
| <http://cathwww.biochem.ucl.ac.uk/cgi-bin/cath/CathedralServer.pl> | CATHEDRAL |  |
| <http://www.ebi.ac.uk/msd-srv/ssm/> | SSM | Secondary structure matching. Also allows multiple structure alignment |
| ***References:*** Assessment of structural alignment methods performance[7]. | | |
| **Molecular visualization software** | | |
| *URL* | *Service name* | *Additional info* |
| <http://www.netsci.org/Resources/Software/Modeling/Viewers/vmd.html> | VMD |  |
| <http://www.astex-therapeutics.com/AstexViewer/AstexViewer2/jsinterface.html> | AstexViewer |  |
| **Identification of cavities** | | |
| *URL* | *Service name* | *Additional info* |
| <http://sts-fw.bioengr.uic.edu/castp/calculation.php> | Functional cavities database | CASTp |
| <http://bmbpcu36.leeds.ac.uk/qsitefinder/help.html> | Q-SiteFinder | Predicts ligand binding sites in a protein structure |
| **Analysis of protein surface electrostatics** | | |
| *URL* | *Service name* | *Additional info* |
| <http://wiki.c2b2.columbia.edu/honiglab_public/index.php/Software:GRASP2> | Analysis of protein surface electrostatics | GRASP |
| **Identification of structural motifs** | | |
| *URL* | *Service name* | *Additional info* |
| <http://portray.bmc.uu.se/cgi-bin/spasm/scripts/spasm.pl> | SPASM/  RIGOR |  |
| <http://sunserver.cdfd.org.in:8080/protease/PAR_3D/index.html> | PAR-3D | Predicts active sites |
| <http://www.russell.embl-heidelberg.de/pints/> | PINTS |  |
| <http://wwwmgs.bionet.nsc.ru/mgs/gnw/pdbsitescan> | PDBSiteScan |  |
| **Prediction of functional residues** | | |
| *URL* | *Service name* | *Additional info* |
| <http://consurf.tau.ac.il/> | ConSurf |  |
| <http://mammoth.bcm.tmc.edu/server.html> | Evolutionary Trace |  |
| <http://sitefinder3d.mbb.yale.edu/> | Site  Finder |3D |  |
| **‘De novo’ prediction of DNA and RNA binding sites** | | |
| *URL* | *Service name* | *Additional info* |
| <http://cubic.bioc.columbia.edu/services/disis> | DISIS | DNA binding |
| <http://bioinfo.ggc.org/bindn> | BindN |  |
| [http://pfp.technion.ac.il](http://pfp.technion.ac.il/) | PatchFinder plus | Needs structure as input |
| **‘De novo’ prediction of metal binding sites** | | |
| *URL* | *Service name* | *Additional info* |
| <http://metaldetector.dsi.unifi.it/help.php> | *METAL DETECTOR* | Uses sequence as input (predicts only histidine and cysteine binding) |
| <http://bioinf.cs.ucl.ac.uk/MetSite/MetSite.html> | MetSite | Needs structure as input |
| <http://ligin.weizmann.ac.il/~lpgerzon/mbs4/mbs.cgi> | CHED | Needs structure as input |
| **‘De novo’ prediction of sub-cellular localization** | | |
| *URL* | *Service name* | *Additional info* |
| <http://cubic.bioc.columbia.edu/services/LOCtree> | LOCtree |  |
| <http://gpcr.biocomp.unibo.it/bacello> | BaCelLO |  |
| <http://www.cbs.dtu.dk/services/TargetP> | TargetP |  |
| [http://pprowler.imb.uq.edu.au](http://pprowler.imb.uq.edu.au/) | Protein Prowler |  |
| [http://psort.nibb.ac.jp](http://psort.nibb.ac.jp/) | PSORT |  |
| ***References:*** A comprehensive review of sub-cellular localization prediction methods [8] | | |
| **Direct predictions of GO terms** | | |
| *URL* | *Service name* | *Additional info* |
| <http://www.cbs.dtu.dk/services/ProtFun> | ProtFun |  |
| [http://proknow.mbi.ucla.edu](http://proknow.mbi.ucla.edu/) | ProKnow | Needs structure as input |
| [http://www.ebi.ac.uk/thornton-srv/databases/ProFunc](http://www.ebi.ac.uk/thornton-srv/databases/ProFunc/) | ProFunc | Needs structure as input |

1. Pearson WR, Sierk ML (2005) The limits of protein sequence comparison? Curr Opin Struct Biol 15: 254-260.

2. Edgar RC, Batzoglou S (2006) Multiple sequence alignment. Curr Opin Struct Biol 16: 368-373.

3. Notredame C (2007) Recent evolutions of multiple sequence alignment algorithms. PLoS Comput Biol 3: e123.

4. Liu J, Rost B (2003) Domains, motifs and clusters in the protein universe. Curr Opin Chem Biol 7: 5-11.

5. Sam V, Tai CH, Garnier J, Gibrat JF, Lee B, et al. (2008) Towards an automatic classification of protein structural domains based on structural similarity. BMC Bioinformatics 9: 74.

6. Kolodny R, Petrey D, Honig B (2006) Protein structure comparison: implications for the nature of 'fold space', and structure and function prediction. Curr Opin Struct Biol 16: 393-398.

7. Kolodny R, Koehl P, Levitt M (2005) Comprehensive evaluation of protein structure alignment methods: scoring by geometric measures. J Mol Biol 346: 1173-1188.

8. Gardy JL, Brinkman FS (2006) Methods for predicting bacterial protein subcellular localization. Nat Rev Microbiol 4: 741-751.
